# Supplementary material for: Long-persisting SARS-CoV-2 spike-specific CD4+ T cells associated with mild disease and increased cytotoxicity post COVID-19
Source: Nat Commun. 2025 Oct 1;16:8743. doi: 10.1038/s41467-025-63711-9 (PMC12489100; doi:10.1038/s41467-025-63711-9)
Supplement: Supplementary file 1 — Supplementary Information [file 41467_2025_63711_MOESM1_ESM.pdf]

Long-persisting SARS-CoV-2 spike-specific CD4$^+$ T cells associated with mild disease and increased cytotoxicity post COVID-19

Supplementary Figures:

Supplementary Fig. 1: HLA-restriction of the three dominant spike epitopes and representative traces of purity checks of T cell clones and bulk lines by peptide-MHC class II tetramers after each round of expansion.

Supplementary Fig. 2: TCR clonotype diversity and similarity of the three dominant spike-specific CD4$^+$ T cell responses.

Supplementary Fig. 3: Association of dominant TCRα clonotypes with COVID-19 disease severity.

Supplementary Fig. 4: Longitudinal TCR clonotype analysis between 1-3 months and 3-4 years after initial infection.

Supplementary Fig. 5: The analysis of patient-specific subpopulations and batch effects on the clustering.

Supplementary Fig. 6: Transcriptomic comparison of spike-specific CD4$^+$ T cells between two timepoints and among different epitopes and cytotoxicity dependency on HLA-DR.

Supplementary Fig. 7: Gating strategy for flow cytometry assays.

Supplementary Tables:

Supplementary Table 1: Clinical characteristics of participants for spike-specific CD4+ T cell responses.

Supplementary Table 2: COVID-19 patients in Meckiff et al. and Bacher et al. dataset

Supplementary Table 3: Genes used to calculate module scores for all single cells in the dataset.

Supplementary Data:

Supplementary Data 1: Clinical characteristics of cohort in this study.

Supplementary Data 2: Public TCRα and TCRβ clonotypes of $S_{166-180}$-, $S_{751-765}$- and $S_{866-880}$-specific T cells identified in our dataset.

Supplementary Figures

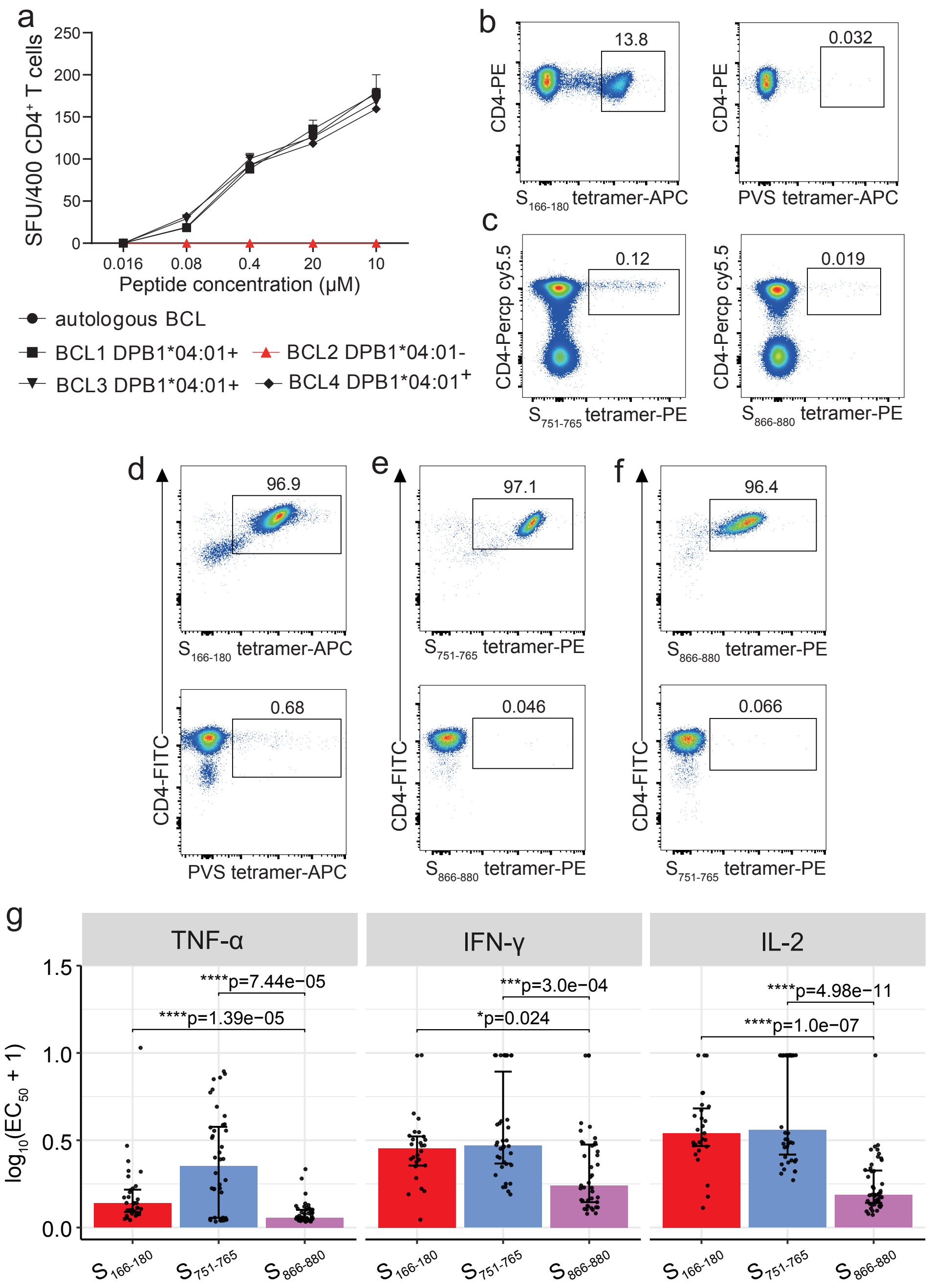

Supplementary Fig. 1: HLA-restriction of the three dominant spike epitopes and representative traces of purity checks of T cell clones and bulk lines by peptide-MHC class II tetramers after each round of expansion. (a) HLA-restriction of $S_{166\text{-}180}$ was identified by $S_{166\text{-}180}$-specific T cell bulk line, which was tested by IFN-γ ELISPOT assay by co-culturing with HLA-matched and -unmatched B cell lines loaded with titrated peptide. **(b)** HLA-restriction of $S_{166\text{-}180}$ was confirmed by peptide-DPB1*04:01-tetramer staining of bulk spike-specific $CD4^{+}$ T cell lines, with PVSKMRMATPLLMQA-DPB1*04:01-tetramer as negative control. **(c)** $S_{751\text{-}765}$ and $S_{866\text{-}880}$ HLA-restriction was confirmed by peptide-DRB1*15:01-tetramer staining of PBMCs from convalescent patients. **(d)** Representative flow cytometry plots of $S_{166\text{-}180}$ -specific T cell clones with corresponding tetramers (top panel) and negative tetramers (bottom panel). **(e)** Representative flow cytometry plots of $S_{751\text{-}765}$-specific T cell clones with corresponding tetramers (top panel) and negative tetramers (bottom panel). **(f)** Representative flow cytometry plots of a $S_{866\text{-}880}$-specific T cell clones with corresponding tetramers (top panel) and negative tetramers (bottom panel). **(g)** Comparison of EC_{50} of cytokine production (TNF-α, IFN-γ and IL-2) upon stimulation of epitope-specific T cell clones (*n*=32 $S_{166\text{-}180}$, *n*=45 $S_{751\text{-}765}$, *n*=48 $S_{866\text{-}880}$) with each of the three spike peptides. Cytokine production was assessed by intracellular cytokine staining. BCL, B cell line.

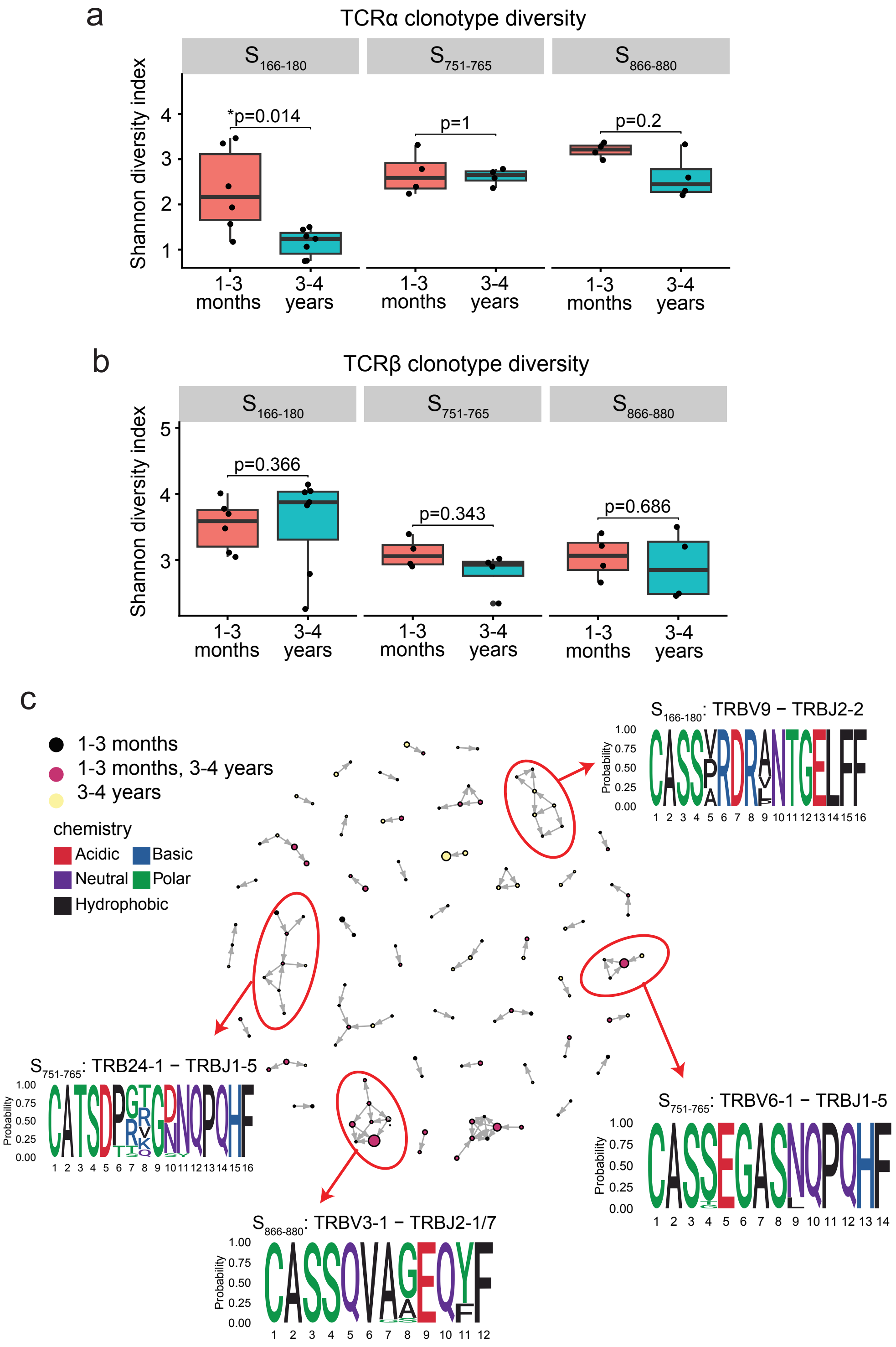

Supplementary Fig. 2: TCR clonotype diversity and similarity of the three dominant spike-specific CD4^{+} T cell responses. (a) Shannon diversity index for TCRα clonotypes for $S_{166\text{-}180}$-, $S_{751\text{-}765}$- and $S_{866\text{-}880}$-specific T cells at 1-3 months (pink bars) and 3-4 years (blue bars) post primary infection. **(b)** Shannon diversity index for TCRβ clonotypes for $S_{166\text{-}180}$-, $S_{751\text{-}765}$- and $S_{866\text{-}880}$-specific T cells at 1-3 months (pink bars) and 3-4 years (blue bars). **(c)** TCRβ clonotype similarity network. Each vertex corresponds to an individual TCR clonotype, with edges connecting vertices if the CDR3 amino acid sequences show a normalised edit distance >0.9 (scRepertoire). The size of the vertex corresponds to the TCR clonotype frequency and colour represents the timepoint they are found at. Cluster motifs were generated using ggseqlogo and amino acids colours based on their biochemical properties.

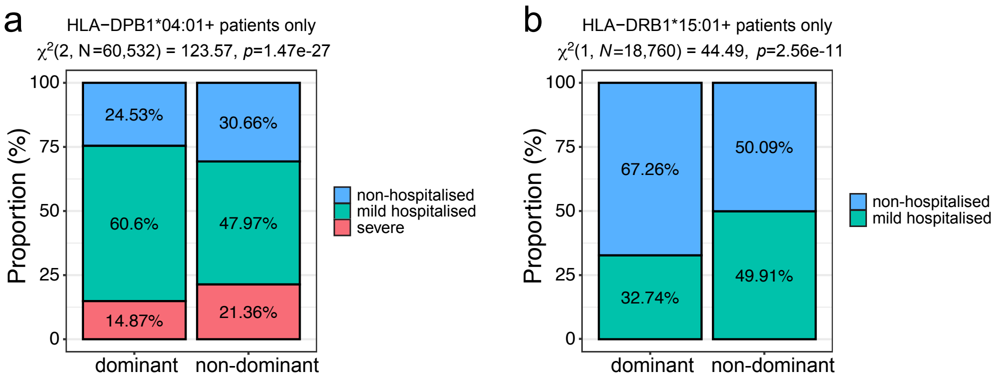

Supplementary Fig. 3: Association of dominant TCRα clonotypes with COVID-19 disease severity. (a) Proportion of cells from HLA-DPB1*04:01+ patients in the Meckiff et al. and Bacher et al. dataset with dominant $S_{166\text{-}180}$-specific and non-dominant TCRα clonotypes. **(b)** Proportion of cells from HLA-DRB1*15:01+ COVID-19 individuals with dominant $S_{751\text{-}765}$ and $S_{866\text{-}880}$-specific and non-dominant TCRα clonotypes.

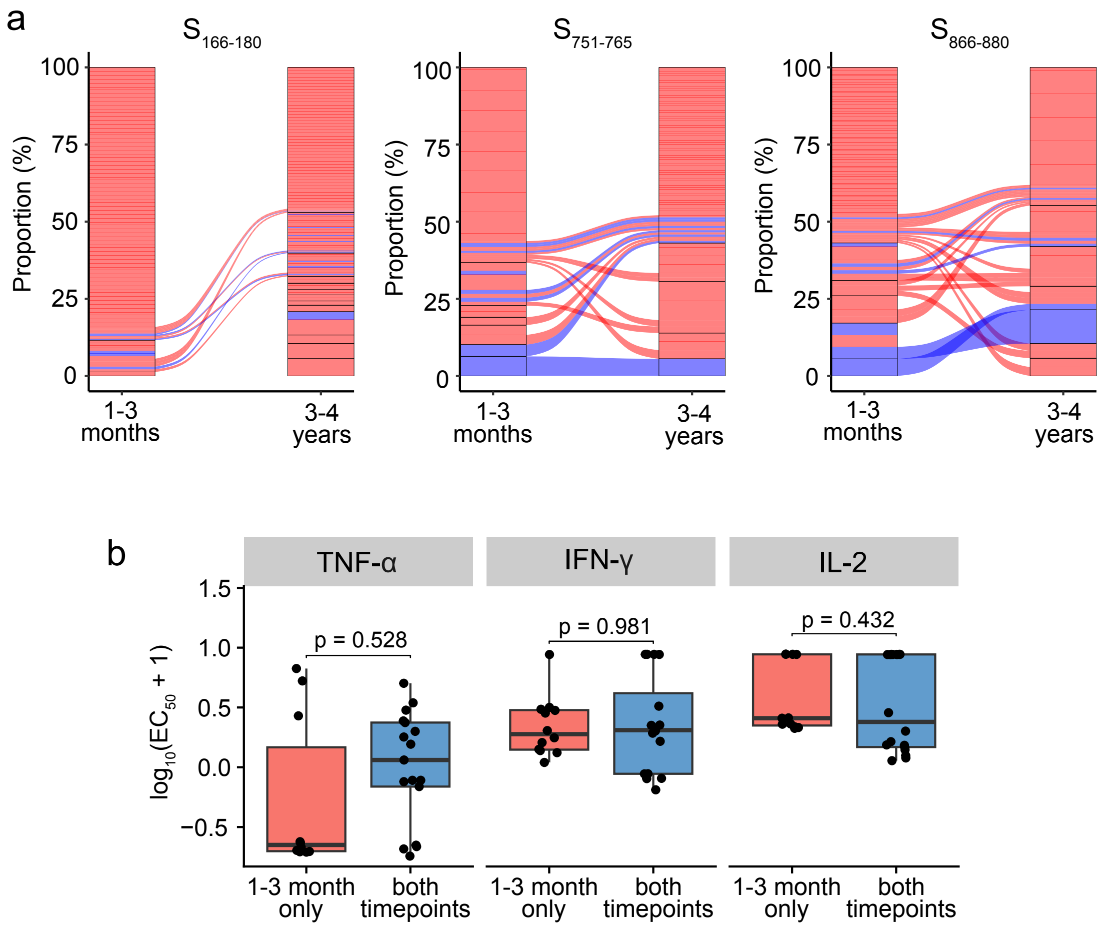

Supplementary Fig. 4: Longitudinal TCR clonotype analysis between 1-3 months and 3-4 years after initial infection. (a) Alluvial plots highlighting the TCRβ clonotypes at 1-3 months and 3-4 years, with links between columns denoting clonotypes found at both timepoints for $S_{166-180}$- (left), $S_{751-765}$- (middle) and $S_{866-880}$-specific (right) T cells. TCRβ clonotypes coloured in blue denote public clonotypes found in more than one individual. **(b)** Comparison of EC_{50} of cytokine production (TNF-α, IFN-γ and IL-2) upon stimulation of $S_{751-765}$-specific T cell clones with TCRα clonotypes found in both 1-3-month and 3-4-year samples (both timepoints, TNF-α *n*=17, IFN-γ *n*=16 and IL-2 *n*=18), or those just found at the 1-3-month timepoint (1-3 month only, TNF-α *n*=10, IFN-γ *n*=12 and IL-2 *n*=11). Cytokine production was assessed by intracellular cytokine staining. Wilcoxon signed-rank test was used to compare between groups and two-sided p-values calculated.

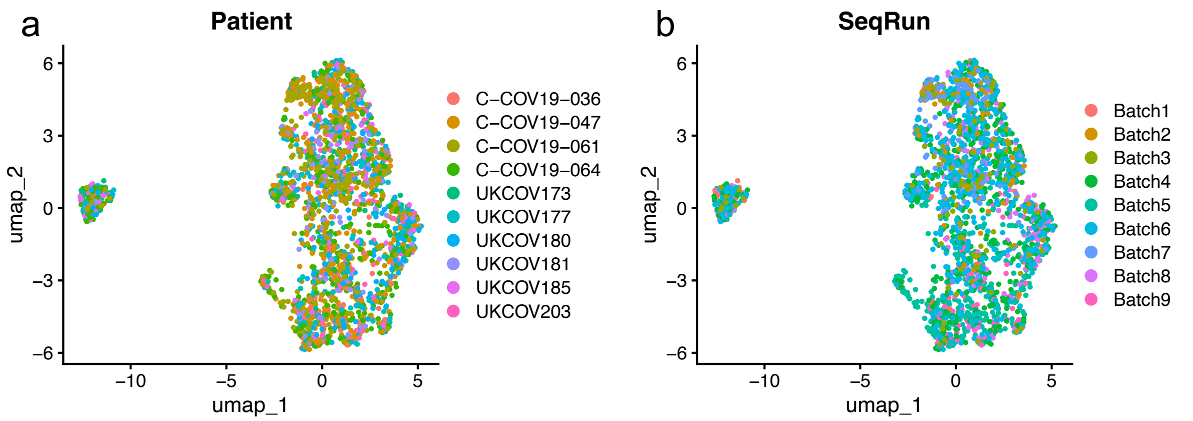

Supplementary Fig. 5: The analysis of patient-specific subpopulations and batch effects on the clustering. **a**, **b**, Uniform manifold approximation and projection (UMAP) visualizations of 2213 cells profiled *ex vivo* from PBMC samples. Cells are coloured based on patients **(a)** and batch of sequencing runs **(b)**.

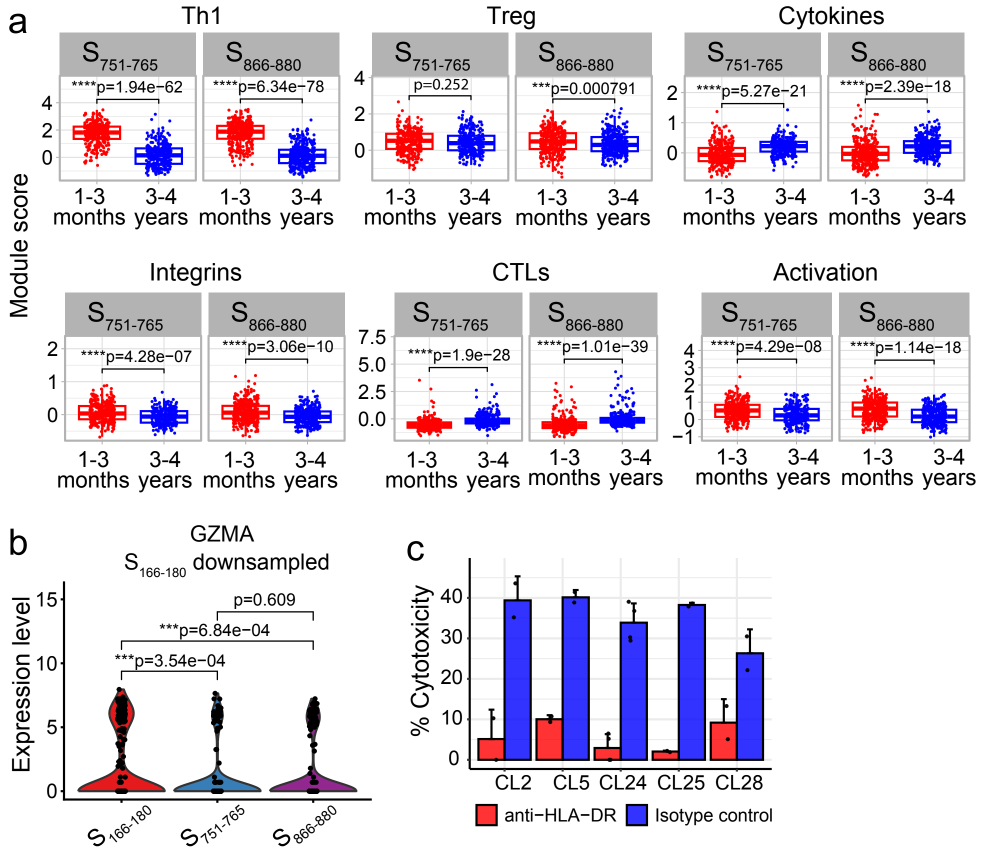

Supplementary Fig. 6: Transcriptomic comparison of spike-specific CD4⁺ T cells between two timepoints and among different epitopes and cytotoxicity dependency on HLA-DR. (a) Boxplots comparing module scores for cells from the 1-3-month and 3-4-year timepoint with analysis carried out separately for the $S_{751\text{-}765}$- and $S_{866\text{-}880}$-specific T cells. **(b)** Violin plots comparing the expression of *GZMA* in cells at 3-4 years between the three epitope-specific cells, with the $S_{166\text{-}180}$-specific cells down-sampled to 300 cells, the average number of cells for the other two epitopes in our dataset. **(c)** Effect of anti-HLA-DR blocking on the cytotoxicity of 5 $S_{866\text{-}880}$ $CD4^+$ T cell clones, which shows marked reduction in cytotoxicity compared to treatment with an isotype control. HLA-DR antibody reduces the cytotoxicity to <10%, resulting in a 10% threshold for determining a cytotoxic clone. Wilcoxon signed-rank test was used to compare between groups.

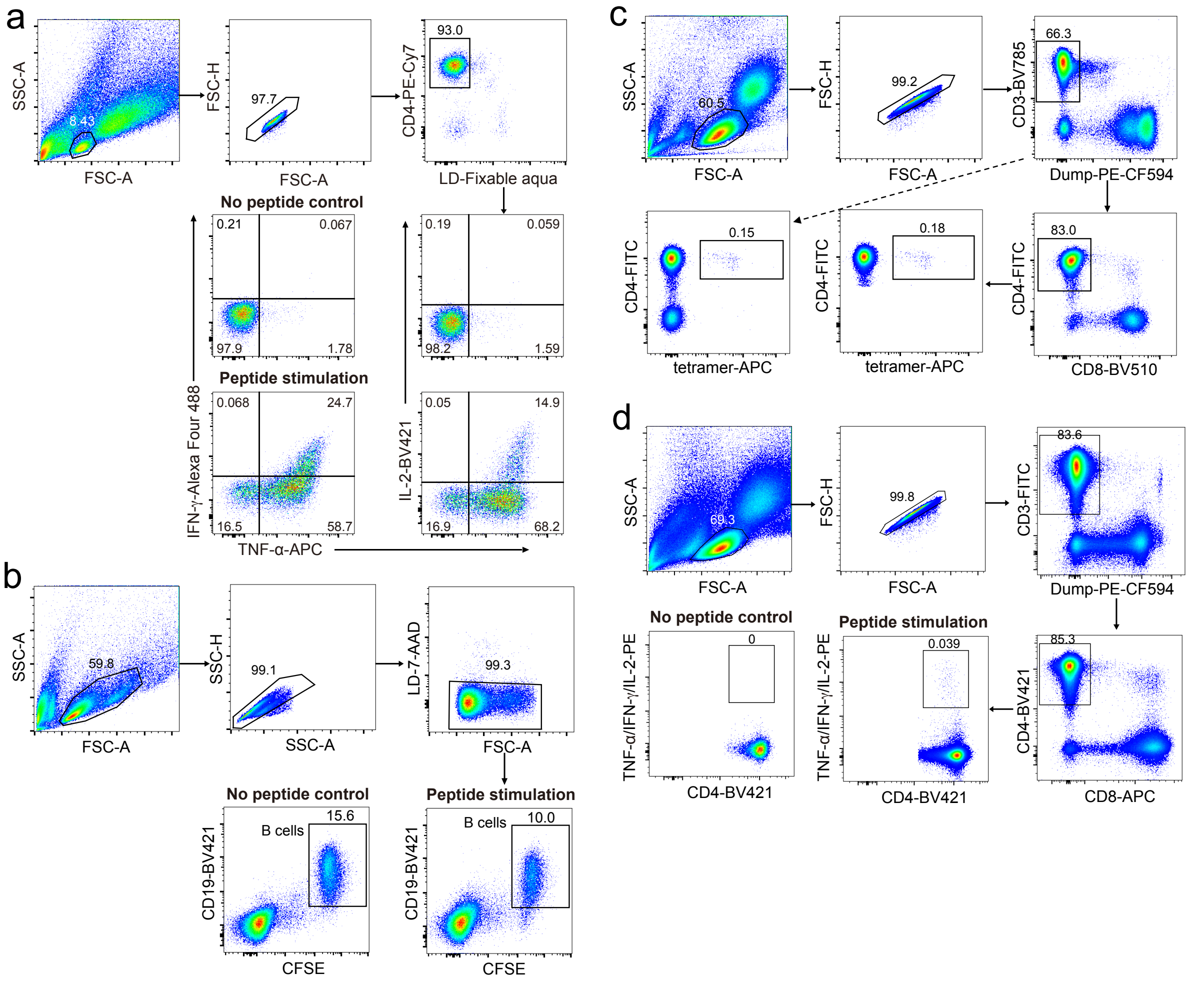

Supplementary Fig. 7: Gating strategy for flow cytometry assays. (a) Representative FACS gating strategy of intracellular cytokine staining (ICS) assay. **(b)** Representative FACS gating strategy of CFSE-based cytotoxic T lymphocyte killing assay. **(c)** Representative FACS gating strategy of single cell sorting of tetramer^{+} CD4^{+} T cells for SmartSeq2 analysis. **(d)** Representative FACS gating strategy of single cell sorting cytokine producing CD4^{+} T cells for SmartSeq2 analysis. Dump in **(c)** and **(d)** includes live dead, CD14, CD16 and CD19.

Supplementary Tables

Supplementary Table 1: Clinical characteristics of participants for spike-specific $CD4^+$ T cell responses.

Participant ID	Severity	$S_{166-180}$		$S_{751-765}$		$S_{866-880}$	
		1-3 months	3-4 years	1-3 months	3-4 years	1-3 months	3-4 years
C-COV19-022	mild	Cytokine-sorted	-	-	-	-	-
C-COV19-035	severe	Cytokine-sorted	-	-	-	-	-
C-COV19-036	severe	-	-	Tetramer-sorted	-	Tetramer-sorted	-
C-COV19-047	mild	Cytokine-sorted	Tetramer-sorted	Tetramer-sorted	Tetramer-sorted	Tetramer-sorted	Tetramer-sorted
C-COV19-064	mild	-	-	Tetramer-sorted	Tetramer-sorted	Tetramer-sorted	Tetramer-sorted
C-COV19-061	severe	-	-	Tetramer-sorted	Tetramer-sorted	Tetramer-sorted	Tetramer-sorted
UKCOV173	severe	-	Tetramer-sorted	-	-	-	-
UKCOV177	critical	-	Tetramer-sorted	-	-	-	-
UKCOV180	critical	Tetramer-sorted	Tetramer-sorted	Tetramer-sorted	Tetramer-sorted	Tetramer-sorted	Tetramer-sorted
UKCOV181	critical	-	-	Tetramer-sorted	Tetramer-sorted	Tetramer-sorted	Tetramer-sorted
UKCOV185	severe	-	Tetramer-sorted	-	-	-	-
UKCOV203	critical	Cytokine-sorted	Tetramer-sorted	-	-	-	-
UKCOV245	critical	Cytokine-sorted	Tetramer-sorted	-	-	-	-

Supplementary Table 2: COVID-19 patients in Meckiff et al. and Bacher et al. dataset.

Meckiff et al.					
ID	Status	DPB1-1	DPB1-2	DRB1-1	DRB1-2
P01	Ward			14:04:01	07:01:01
P03	Ward			14:54:01	15:01:01
P04	Ward				
P05	ICU				
P06	Ward			01:01:01	03:01:01
P07	Ward			14:54:01	07:01:01
P0901	Ward	04:01:01	04:01:01	15:01:01	16:01:01
P10	ICU	18:01:01	01:01:01	07:01:01	15:03:01
P12	Ward	1321:01:00	04:01:01	15:01:01	11:04:01
P15	Ward				
P16	Ward	09:01:01	04:02:01	07:01:01	15:02:01
P17	Ward	1321:01:00	03:01:01	01:01:01	13:02:01
P18	Ward			04:01:01	07:01:01
P19	Ward			15:01:01	14:54:01
P20	ICU	04:01:01	04:02:01	11:03:01	13:01:01
P22	mild	1321:01:00	04:01:01	04:03:01	11:04:01
P24	ICU	04:02:01	1068:01:00	04:01:01	08:01:01
P25	mild			07:01:01	04:04:01
P26	mild				
P27	ICU				
P29	mild				
P30	mild			15:01:01	14:54:01
P31	mild				
P32	mild				
P37	mild				
P40	mild			01:01:01	04:01:01
P42	ICU			15:06:01	01:01:01
P43	ICU			13:36	04:05:01
P44	mild	04:01:01	10:01:02	12:01:01	11:01:01
P45	mild				
P46	ICU	01:01:01	06:01:01	07:01:01	03:01:01
P47	mild				
P49	ICU			15:06:01	03:01:01
P57	mild				
P61	mild				
P64	mild				
P66	mild			09:01:02	03:01:01
Bacher et al.					
ID	Status	DPB1-1	DPB1-2	DRB1-1	DRB1-2
J10535	mild	05:01:01	04:01:01	01;01:01	03:01:01
J10886	mild	02:01:02	04:01:01	07:01:01	08:02:01
J10888	mild	04:01:01	04:02:01	07:01:01	11:01:01
J14205	mild	02:01:02	04:01:01	04:03:01	01:01:01
J15893	mild	04:01:01	04:01:01	08:01:01	11:01:01
J09835	non-hospitalised	04:01:01	04:02:01	07:01:01	15:01:01
J09836	non-hospitalised	02:01:02	04:01:01	11:01:01	15:01:01
J10624	non-hospitalised	04:01:01	04:01:01	15:01:01	15:01:01
J10625	non-hospitalised	02:01:02	04:01:01	01:01:01	15:01:01
J11689	non-hospitalised	04:01:01	01:01:01	03:01:01	15:01:01
J15890	non-hospitalised	02:01:02	04:01:01	04:01:01	13:01:01
J10887	severe	14:01:01	04:02:01	14:54:01	04:01:01
J14204	severe	02:01:02	02:01:02	13:02:01	11:04:01
J21854	severe	04:01:01	04:02:01	04:01:01	13:02:01

Supplementary Table 3: Genes used to calculate module scores for all single cells in the dataset.

Positive regulation of Th1 differentiation	Treg	Cytokines	Integrins	Cytotoxicity	Activation
ANXA1	FOXP3	IFNG	SELPLG	GZMA	CD44
CCL19	CCR7	TNF	ICAM1	GZMB	CD27
CCR2	SELL	IL23A	ITGA10	GZMH	CD28
CCR7	IL7R	IL24	ITGAE	GZMK	CD69
HLX	PRDM1	IL16	ITGB3	GZMM	CD38
IRF1	IL2RA	IL32	CD107A	PRF1	HLA-DRB1
RIPK2	STAT5A	IL2	ITGB7	GNLY	TUBA1B
SOCS5	STAT5B	IL6	ITGAL	NKG7	TNGRSF9
	TGFB1	CSF2	ITGAD		IL3RA
	IL10	IL13	ITGB2		RNFSF4
	IL35	CCL3	ITGA9		
		CCL4	ITGB1		
		CCL5	ITGB5		
		CCL20	ITGA10		
		CCL4L2	ITGB8		
			ITGA2		
			ITGA4		
			ITGA5		
			ITGA6		
			ITGB4		
			ITGA11		
			ITGAV		
			ITGAM		
			ITGAX		
			ITGA2B		
			ITGA3		
			ITGA8		
