# Supplementary material for: Long-persisting SARS-CoV-2 spike-specific CD4+ T cells associated with mild disease and increased cytotoxicity post COVID-19
Source: Nat Commun. 2025 Oct 1;16:8743. doi: 10.1038/s41467-025-63711-9 (PMC12489100; doi:10.1038/s41467-025-63711-9)
Supplement: Supplementary file 2 — Description of Additional Supplementary Files [file 41467_2025_63711_MOESM2_ESM.pdf]

Description of Additional Supplementary Files

Supplementary Data 1: Characteristics of the individuals investigated in this study, highlighting sex, age range, disease severity, sampling timepoints and MHC class II alleles. M, male; F, female.

Supplementary Data 2: Public TCRα/β clonotypes in our scRNA-seq data. Clonotypes are defined as CDR3 amino acid sequence + V gene usage, with public clonotypes found in >1 unrelated individuals. CDR3, complementarity determining region 3; aa, amino acid; V gene, variable gene; J gene, joining gene.
