# Supplementary material for: Long-persisting SARS-CoV-2 spike-specific CD4+ T cells associated with mild disease and increased cytotoxicity post COVID-19
Source: Nat Commun. 2025 Oct 1;16:8743. doi: 10.1038/s41467-025-63711-9 (PMC12489100; doi:10.1038/s41467-025-63711-9)
Supplement: Supplementary file 4 — Reporting Summary [file 41467_2025_63711_MOESM4_ESM.pdf]

nature portfolio

Corresponding author(s): Tao Dong

Last updated by author(s): Jul 30, 2025

Reporting Summary

Nature Portfolio wishes to improve the reproducibility of the work that we publish. This form provides structure for consistency and transparency in reporting. For further information on Nature Portfolio policies, see our Editorial Policies and the Editorial Policy Checklist.

Statistics

For all statistical analyses, confirm that the following items are present in the figure legend, table legend, main text, or Methods section.

n/a	Confirmed	
☐	☒	The exact sample size (n) for each experimental group/condition, given as a discrete number and unit of measurement
☐	☒	A statement on whether measurements were taken from distinct samples or whether the same sample was measured repeatedly
☐	☒	The statistical test(s) used AND whether they are one- or two-sided<br>*Only common tests should be described solely by name; describe more complex techniques in the Methods section.*
☐	☒	A description of all covariates tested
☐	☒	A description of any assumptions or corrections, such as tests of normality and adjustment for multiple comparisons
☐	☒	A full description of the statistical parameters including central tendency (e.g. means) or other basic estimates (e.g. regression coefficient) AND variation (e.g. standard deviation) or associated estimates of uncertainty (e.g. confidence intervals)
☐	☒	For null hypothesis testing, the test statistic (e.g. F, t, r) with confidence intervals, effect sizes, degrees of freedom and P value noted<br>*Give P values as exact values whenever suitable.*
☒	☐	For Bayesian analysis, information on the choice of priors and Markov chain Monte Carlo settings
☒	☐	For hierarchical and complex designs, identification of the appropriate level for tests and full reporting of outcomes
☒	☐	Estimates of effect sizes (e.g. Cohen's d, Pearson's r), indicating how they were calculated

Our web collection on statistics for biologists contains articles on many of the points above.

Software and code

Policy information about availability of computer code

Data collection	ELISpot data were collected with AID ELISpot 7.0 and flow cytometry data were collected by BD FACSDiva V9.0/ Attune™ NxT software V3.2.1 Sequencing data were collected with NextSeq Control Software (NCS) version 4 or MiSeq Control Software version 2.6.2.1 or Nava Seq Control Softmare version 1.7.5. Proteomics data were collected with Orbitrap Fusion Lumos Control Software version 3.3.
Data analysis	Flow cytometry data were analyzed with FlowJo v.10.10.0 software for MacOS. Raw BCL files from SmartSeq2 single cell RNA sequencing were converted to FASTQ format using bcl2fastq (v 2.20.0.422). FASTQ files were aligned to human genome hg19 using STAR (v 2.6.1D). Reads were counted using featureCounts (part of subread v 2.0.0). Resulting count matrices were analysed in R v 4.0.1 using Seurat (v 3.9.9.9010). Packages used in R for additional analyses or data visualisation are: Harmony (v 1.0), ggplot2 (v 3.3.2), tcR (v 2.3.2), circlize (v 0.4.12), stringdist (v 0.9.6), ggalluvial (v 0.12.2), pheatmap (v 1.0.12). TCR sequences were reconstructed from FASTQ files using MiXCR (v 3.0.13). The DIA raw mass spec data from proteomics was analyzed using the label free quantitation software (LFQ) called DIA-NN using the library free approach.

For manuscripts utilizing custom algorithms or software that are central to the research but not yet described in published literature, software must be made available to editors and reviewers. We strongly encourage code deposition in a community repository (e.g. GitHub). See the Nature Portfolio guidelines for submitting code & software for further information.

Data

Policy information about availability of data

All manuscripts must include a data availability statement. This statement should provide the following information, where applicable:

- Accession codes, unique identifiers, or web links for publicly available datasets
- A description of any restrictions on data availability
- For clinical datasets or third party data, please ensure that the statement adheres to our policy

The raw data from all the main figures and supplementary figures are provided in a Source Data file. The single-cell RNA-seq data generated in this study has been deposited in the ArrayExpress database under accession code E-MTAB-14933. The mass spectrometry proteomics data has been deposited to the ProteomeXchange Consortium via the PRIDE partner repository with the dataset identifier PXD042469.

Research involving human participants, their data, or biological material

Policy information about studies with human participants or human data. See also policy information about sex, gender (identity/presentation), and sexual orientation and race, ethnicity and racism.

Reporting on sex and gender	Sex was recorded in Supplementary Table 1 but not considered in study design; sex was determined based on sef-reporting. No sex-based analysis has been performed due to low sample size.
Reporting on race, ethnicity, or other socially relevant groupings	Socially constructed or socially relevant categorization variables were not used in this manuscript.
Population characteristics	48 individuals were recruited following recovery from COVID-19. Supplementary Table 1 shows the participant characteristics.
Recruitment	Participants were recruited from the John Radcliffe Hospital in Oxford, UK, between March 2020 and September2021 by identification of individuals hospitalised during the SARS-COV-2 pandemic. Participants were recruited into the Sepsis Immunomics study and had samples collected during their convalescence as well as 3-4 year follow-up. For convalescence, participants were sampled at least 28 days from the start of their symptoms. Written informed consent was obtained from all individuals.
Ethics oversight	Ethical approval was given by the South Central-Oxford C Research Ethics Committee in England (ref. 19/SC/0296).

Note that full information on the approval of the study protocol must also be provided in the manuscript.

Field-specific reporting

Please select the one below that is the best fit for your research. If you are not sure, read the appropriate sections before making your selection.

☒ Life sciences ☐ Behavioural & social sciences ☐ Ecological, evolutionary & environmental sciences

For a reference copy of the document with all sections, see nature.com/documents/nr-reporting-summary-flat.pdf

Life sciences study design

All studies must disclose on these points even when the disclosure is negative.

Sample size	48 individuals recovered from COVID-19 samples. This was a follow-up for a published study (Peng et al, NI, https://doi.org/10.1038/s41590-020-0782-6). Samples sizes were based on maximal available samples sets where detailed clinical and serological data were also available, and aligned well with our previously published data in other settings, including influenza and HIV (Lee et al, JCI 2008; Zhang et al, NC 2013; Zhao et al, AJCCM 2012)
Data exclusions	For ELISpot assays, If negative control wells had >30 SFU/106 PBMCs or positive control wells (PHA stimulation) were negative, the results were excluded from further analysis. There were no data excluded from ELISpot analysis.<br>For scRNA-Seq analysis cells were filtered using the following criteria: minimum number of cells expressing specific gene = 3, minimum number of genes expressed by cell = 200 and maximum number of genes expressed by cell = 4000. Cells were also excluded if they expressed more than 5% mitochondrial genes.<br>For paired αβ chain TCR analysis, cells were filtered to retain only 1α1β or 2α1β cells.<br>For single β chain TCR analysis, cells were filtered to retain only 1β (regardless of number of α) to use for downstream analysis.
Replication	Samples analyzed in this study were from participants of a cohort study and samples were analyzed on individual study participants.<br>Ex vivo experiments did not include replicates, but results from each participant were confirmed by at least two different experiments.<br>For in vitro assays, results shown were always two or more independent experiments in which every repeat gave similar results.<br>Bulk RNA-Seq and proteomics included three technical replicates for each biological samples.

Randomization

Randomization was not appropriate for this study of immune responses in COVID-19 convalescent individuals, with no associated therapeutic intervention.

Blinding

Blinding was not appropriate for this study of immune responses in COVID-19 convalescent individuals, with no associated therapeutic intervention.

Reporting for specific materials, systems and methods

We require information from authors about some types of materials, experimental systems and methods used in many studies. Here, indicate whether each material, system or method listed is relevant to your study. If you are not sure if a list item applies to your research, read the appropriate section before selecting a response.

Materials & experimental systems

n/a	Involved in the study
☐	☒ Antibodies
☐	☒ Eukaryotic cell lines
☒	☐ Palaeontology and archaeology
☒	☐ Animals and other organisms
☒	☐ Clinical data
☒	☐ Dual use research of concern
☒	☐ Plants

Methods

n/a	Involved in the study
☒	☐ ChIP-seq
☐	☒ Flow cytometry
☒	☐ MRI-based neuroimaging

Antibodies

Antibodies used

Antibodies used for flow cytometry

Marker	Fluorophore	Supplier	Cat number	Clonotype	Lot number	Dilution
CD14	BV510	BioLegend	367124	63D3	B280762	1:50
CD16	BV510	BioLegend	302048	3G8	B289732	1:50
CD19	BV510	BioLegend	302242	HIB19	B281769	1:50
CD19	BV421	BioLegend	302234	HIB19	B342418	1:50
CD14	PE-CF594	BioLegend	367134	3D3	B268423	1:50
CD16	PE-CF594	BioLegend	302054	3G8	B274232	1:50
CD19	PE-CF594	BioLegend	302252	HIB19	B277039	1:50
CD3	BV785	BioLegend	317330	OKT3	B255880	1:33
CD4	FITC	BD Bioscience	347413	SK3/SK4	0349302	1:25
CD4	APC	BD Bioscience	345771	SK3	1111911	1:33
CD4	PE	BD Bioscience	345769	SK3	0335661	1:50
IFN-γ	AF488	BD Bioscience	557718	B27	1299826	1:33
IFN-γ	PE-Cy7	BD Bioscience	557643	B27	1046592	1:50
TNF-α	APC	eBioscience	17-7349-82	Mab11	2330486	1:500
IL-2	BV421	BioLegend	500328	MQ1-17H12	B320878	1:33
ACE2	Primary	R&D	AF933	Polyclonal	HOK0620051	1:20
anti-goat	AF647	AbCam	ab150135		GR3324428-3	1:1000
CD3	FITC	BD Bioscience	340542	SK7	0106592	1:20
CD4	BV421	BioLegend	344632	SK3	B321520	1:50
CD8	BV421	BioLegend	344748	SK1	B331004	1:50
CD8	APC	BD Bioscience	340584	SK1	0162290	1:50
CD8	BV510	BioLegend	344732	SK1	B347289	1:33
TNF-α	PE	Miltenyi Biotec	130-091-268		5230908464	1:10
IFN-γ	PE	Miltenyi Biotec	130-054-202		5230705554	1:10
IL-2	PE	Miltenyi Biotec	130-090-487		5230904162	1:10

Validation

All antibodies used in this study are commercially available. Antibodies used in a specific species or application have been appropriately validated by manufacturers for that application and this information is provided on their website and product information datasheets. All antibodies described here have been further optimized for an appropriate concentration by testing several dilutions.
https://www.biolegend.com/en-gb/products/brilliant-violet-510-anti-human-cd14-antibody-14983?Clone=63D3
https://www.biolegend.com/en-gb/products/brilliant-violet-510-anti-human-cd16-antibody-8003?Clone=3G8
https://www.biolegend.com/en-gb/products/brilliant-violet-510-anti-human-cd19-antibody-8004?Clone=HIB19
https://www.biolegend.com/en-gb/products/brilliant-violet-421-anti-human-cd19-antibody-7144?Clone=HIB19
https://www.biolegend.com/en-gb/products/pedazzle-594-anti-human-cd14-antibody-16540?Clone=63D3
https://www.biolegend.com/en-gb/products/pe-dazzle-594-anti-human-cd16-antibody-9810?Clone=3G8
https://www.biolegend.com/en-gb/products/pe-dazzle-594-anti-human-cd19-antibody-9783?Clone=HIB19
https://www.biolegend.com/en-gb/products/brilliant-violet-785-anti-human-cd3-antibody-7977?Clone=OKT3
https://www.bdbiosciences.com/en-gb/products/reagents/flow-cytometry-reagents/clinical-discovery-research/multicolor-cocktails-and-kits-ruo-gmp/fitc-mouse-anti-human-cd4.347413
https://www.bdbiosciences.com/en-gb/products/reagents/flow-cytometry-reagents/clinical-diagnostics/single-color-antibodies-asr-ivd-ce-ivd/cd4-apc.345771

https://www.bdbiosciences.com/en-gb/products/reagents/flow-cytometry-reagents/clinical-diagnostics/single-color-antibodies-asr-ivd-ce-ivd/cd4-pe.345769
https://www.bdbiosciences.com/en-gb/products/reagents/flow-cytometry-reagents/research-reagents/single-color-antibodies-ruo/alexa-fluor-488-mouse-anti-human-ifn.557718
https://www.bdbiosciences.com/en-gb/products/reagents/flow-cytometry-reagents/research-reagents/single-color-antibodies-ruo/pe-cy-7-mouse-anti-human-ifn.557643
https://www.thermofisher.com/antibody/product/TNF-alpha-Antibody-clone-MAb11-Monoclonal/17-7349-82
https://www.biolegend.com/en-gb/products/brilliant-violet-421-anti-human-il-2-antibody-7148?Clone=MQ1-17H12
https://www.rndsystems.com/products/human-mouse-rat-hamster-ace-2-antibody_af933
https://www.abcam.com/donkey-goat-igg-hl-alexa-fluor-647-ab150131.html
https://www.bdbiosciences.com/en-us/products/reagents/flow-cytometry-reagents/clinical-diagnostics/single-color-antibodies-asr-ivd-ce-ivd/cd3-fitc.340542
https://www.biolegend.com/en-gb/products/brilliant-violet-421-anti-human-cd4-antibody-12068
https://www.biolegend.com/en-gb/products/brilliant-violet-421-anti-human-cd8-antibody-13512
https://www.bdbiosciences.com/en-us/products/reagents/flow-cytometry-reagents/clinical-discovery-research/single-color-antibodies-ruo-gmp/apc-mouse-anti-human-cd8.340584
https://www.biolegend.com/en-gb/products/brilliant-violet-510-anti-human-cd8-antibody-10739
https://www.miltenyibiotec.com/GB-en/products/tnf-a-secretion-assay-detection-kits-human.html#130-091-268
https://www.miltenyibiotec.com/GB-en/products/ifn-g-secretion-assay-detection-kits-human.html#130-054-202
https://www.miltenyibiotec.com/GB-en/products/il-2-secretion-assay-detection-kits-human.html#130-090-487

Eukaryotic cell lines

Policy information about cell lines and Sex and Gender in Research

Cell line source(s)	All the EBV-transformed B cell lines were established in the lab. Vero E6 cells (ATCC CCL-81), HEK293T cells (ATCC, CRL-11268) were from ATCC. Vero E6/TMPRSS2 cells were originally from NIBSC (reference 100978).
Authentication	Cell lines were validated by morphology. Vero E6 cells, HEK293T cells were obtained from ATCC and used at low passage. They were not further authenticated. Vero E6/TMPRSS2 from NIBSC were used for propagating SARS-CoV-2 virus and were not further authenticated. EBV transformed B cell lines expressing ACE2 were verified by flow cytometry staining of ACE2 and CD19.
Mycoplasma contamination	All the cell lines were tested negative for mycoplasma .
Commonly misidentified lines (See ICLAC register)	No misidentified cell lines were used according to the version 11 of register of misidentified cell lines.

Plants

Seed stocks	*Report on the source of all seed stocks or other plant material used. If applicable, state the seed stock centre and catalogue number. If plant specimens were collected from the field, describe the collection location, date and sampling procedures.*
Novel plant genotypes	*Describe the methods by which all novel plant genotypes were produced. This includes those generated by transgenic approaches, gene editing, chemical/radiation-based mutagenesis and hybridization. For transgenic lines, describe the transformation method, the number of independent lines analyzed and the generation upon which experiments were performed. For gene-edited lines, describe the editor used, the endogenous sequence targeted for editing, the targeting guide RNA sequence (if applicable) and how the editor was applied.*
Authentication	*Describe any authentication procedures for each seed stock used or novel genotype generated. Describe any experiments used to assess the effect of a mutation and, where applicable, how potential secondary effects (e.g. second site T-DNA insertions, mosiacism, off-target gene editing) were examined.*

Flow Cytometry

Plots

Confirm that:

☒ The axis labels state the marker and fluorochrome used (e.g. CD4-FITC).

☒ The axis scales are clearly visible. Include numbers along axes only for bottom left plot of group (a 'group' is an analysis of identical markers).

☒ All plots are contour plots with outliers or pseudocolor plots.

☒ A numerical value for number of cells or percentage (with statistics) is provided.

Methodology

Sample preparation	For cell sorting for single-cell RNA-seq from 1-3-month samples, cryopreserved PBMCs were thawed and rested overnight in R10 at 37°C. On the second day, thawed PBMCs were stimulated with peptide of S166-180 at a final concentration of 10µM for 5 hours. Then, cells were washed and incubated with TNF-α, IFN-γ, and IL-2 catching antibody for 45 mins, followed by staining with PE-conjugated TNF-α, IFN-γ, and IL-2 detection antibodies (Miltenyi Bio), CD3-BV785 (BioLegend), CD4-APC, CD14-PE-CF594, CD19-PE-CF594 and CD16-PE-CF594 (BD Biosciences). Before sorting, cells were stained with Propidium

Iodide (PI) (eBioscience) to exclude nonviable cells. S751-765- and S866-880- specific CD4+ T cells were sorted with peptide-MHC-class-II tetramers. In brief, 1-3 million cells were stained with APC-conjugated HLA-DRB1*15:01 S751-765 and S866-880 tetramers (ProImmune) respectively. Live/dead fixable Aqua dye (Invitrogen) was used to exclude nonviable cells from the analysis. Cells were washed and stained with the following surface antibodies: CD4-PE (BD Biosciences), CD3-BV785, CD14-BV510, CD19-BV510 and CD16-BV510 (BioLegend). After exclusion of nonviable/CD14+/CD19+/CD16+cells, CD3+CD4+TNF-α +/IFN-γ+/IL-2+ cells or CD3+CD4+Tetramer+ were sorted for scRNA-seq using a BD Fusion sorter or BD FACS Aria III (BD Biosciences). Single cells were directly sorted into 96-well PCR plates (Thermo Fisher Scientific) and stored at −80°C for further SmartSeq2 analysis.
For 3-4-year samples, 15-30 million PBMCs were labelled with APC- or PE-conjugated peptide-MHC-class-II tetramers for 30 minutes. Enrichment was then performed with anti-APC or anti-PE microbeads using magnetic-activated cell sorting technology (Miltenyi Biotec) following the manufacturer's instructions. Subsequently, enriched S166-180-, S751-760- and S866-880-specific CD4+ T cells were stained with CD3-BV786 and CD8-BV510 (BioLegend), CD4-FITC, CD14-PE-CF594, CD19-PE-CF594 and CD16-PE-CF594 (BD Biosciences). Before sorting, cells were stained with Propidium Iodide (PI) (eBioscience) to exclude nonviable cells. CD3+CD8-CD4+tetramer+ were sorted for scRNA-seq using a BD FACSAria Fusion sorter or BD FACS Aria III (BD Biosciences).
For intracellular cytokine staining (ICS), T cells were cocultured with B cells loaded with peptide at 37°C for 6 hours with GolgiPlug and GolgiStop. Cells were stained with Live/Dead Fixable Aqua dye (Invitrogen), followed by surface staining with CD4-PE-Cy7 (BD, Biosciences). After subsequent permeabilisation with Fixation/Permeabilisation solution (BD, Biosciences), cells were stained with including IFN-γ-AF488 (BD Biosciences), TNFα-APC (eBioscence) and IL2-BV421 (BioLegend). Then, washed twice with 1X perm/wash buffer. Finally, cells then were fixed with 1X cell fixing buffer.
For CFSE-based cytotoxic T lymphocyte killing assay, EBV-transformed BCLs were labelled with 0.5µM carboxyfluoroscein succinimidyl ester (CFSE, Thermo Fisher Scientific), then loaded with 2µM of peptide at 37°C for 1 hour. Subsequently, cells were washed, counted and cocultured with T cells at an E:T ratio of 4:1 at 37°C for 6 hours. Samples were then stained with 7-AAD (eBioscience) and CD19-BV421 (BioLegend). To assess the MHC-class II-dependence of the killing, B cell lines were treated with either 40ug/ml of anti-HLA-DR antibody (BioLegend) or isotype control (BioLegend) at room temperature for 1 hour prior to being loaded with peptide. Cell death was assessed based on the presence of CFSE+CD19+7-AAD- (live) cells. Negative controls containing BCLs without peptide pulse and T cells were included for each sample.

Instrument

Samples were sorted using BD Fusion or BD FACS Aria III (BD Biosciences) or acquired at Thermo Fisher Attune™ NxT Flow Cytometer.

Software

Data were collected using FACS DIVA (v9.0.1) or Attune™ NxTsoftware v3.2.1 and analyzed using FlowJo v.10.10.0 software for MacOS.

Cell population abundance

All sorted samples were checked for after-sorting purity (>99%).

Gating strategy

For all the experiments, cells were first gated on single lymphocytes by a forward side scatter gate. On sorting for RNASeq, after excluding dead cells, CD14+, CD19+, and CD16+ cells, S166-180, S751-765 and S866-880-specific T cells were identified as CD3+CD8-CD4+TNF-α/IFN-γ/IL-2+ or CD3+CD8-CD4+tetramer+ . For intracellular cytokine staining (ICS), the cytokine positive/ negative population were gated according to corresponding negative controls, known as unstimulated samples (T cells co-cultured with target cells without peptide loading or without virus infection): after excluding dead cells, cells then were gated into CD4+ T cells TNF-α+/-, IFN-γ+/- and IL-2+/- populations were gated in consistence with the corresponding negative controls. For CFSE-based cytotoxic T lymphocyte killing assay, after excluding dead cells, cells were then gated on B cells with CD19+CFSE+.

☒ Tick this box to confirm that a figure exemplifying the gating strategy is provided in the Supplementary Information.
